# Quality of Life After Myocardial Infarction as a Function of Temperamental Traits, Stress Coping Styles, and Posttraumatic Stress Disorder Symptoms

**DOI:** 10.3389/fpsyt.2021.696544

**Published:** 2022-01-05

**Authors:** Agnieszka Burnos, Maria Wrzosek

**Affiliations:** Faculty of Psychology, University of Warsaw, Warsaw, Poland

**Keywords:** quality of life, myocardial infarction, temperamental traits, stress coping styles, PTSD

## Abstract

The presented study aims to explore the relationship between quality of life after myocardial infarction and factors such as temperamental traits, stress coping, and posttraumatic stress disorder (PTSD) symptoms. Ninety-six participants, including 51 females and 45 males aged 24–85 years, who have survived ST-elevation myocardial infarction were asked to complete the Formal Characteristics of Behavior—Temperament Inventory, Coping Inventory for Stressful Situations, PTSD Inventory, and Quality of Life SF-36 questionnaire. According to the obtained results, a lower level of briskness and sensory sensitivity, as well as a higher level of perseverance and endurance, correlates with a higher level of emotion-oriented coping, whereas a higher level of perseverance, endurance, and activity correlates with a higher level of avoidant-distracted coping. Moreover, a higher level of briskness and activity is correlated with a higher level of avoidant-social coping. A higher level of emotion-oriented and avoidant-distracted coping is, in turn, associated with a higher intensity of PTSD symptoms, whereas a higher level of avoidant-social coping correlates with lower intensity of PTSD symptoms. Furthermore, a higher level of avoidant-distracted coping is correlated to a better physical quality of life, whereas higher levels of endurance and activity are associated with a better emotional quality of life. Also, the more severe the PTSD symptoms, the lower quality of life in general. Contrastingly, higher sensory sensitivity and briskness correlate with better quality of life. The meaning of other temperamental traits, however, is more ambiguous. Nevertheless, the findings support the model of psychological processes in which the subsequent stages are temperament, coping, PTSD, and quality of life.

## Introduction

As a result of medical advances, it is now possible to treat diseases that, such as cardiovascular diseases, were formerly considered fatal. People formerly considered terminally ill can survive with chronic diseases for many years. This phenomenon results in new problems, e.g., stress related to one's state of health, a necessity for both emotional and cognitive adaptation to the illness, its psychological effects, and treatment. There are also some inevitable changes in the family and the social background of people with chronic diseases.

A systematic increase in the number of effectively treated patients can be observed. Therefore, it is essential to address and understand the matters of their emotional and spiritual condition and their professional, social, and family situation. A key aspect is an issue concerning adaptation to the challenges of the new circumstances resulting in a changed level of the quality of life.

To fulfill this demand, it is vital to abandon the unidimensional medical model in favor of the biopsychosocial one, which accentuates the physical, psychological, and social integrity of the human being.

The research presented in this paper aims to explore whether the quality of life after myocardial infarction (MI) and psychological variables, including temperamental characteristics, ways of coping with stress, and symptoms of posttraumatic stress disorder (PTSD), are actually related.

In fact, aside from being a medical phenomenon, MI comes as a highly distressing and unexpected event (1). The affected person would often be unaware of what is happening, experience excruciating chest pain and shortness of breath, become anxious, and fear death (2, 3). Such an experience may indeed be considered a traumatic event, and symptoms of PTSD may subsequently develop (4, 5).

In most cases, the period of hospitalization after MI is not long. However, afterward, patients may have to cope with restrictions in their physical functioning, i.e., they easily become tired and breathless, have to change their diet, and limit their working hours. Although, initially, they tend to be fearful of another heart attack, the level of their anxiety decreases over time (6).

Higher quality of life level along with the absence of posttraumatic symptoms can be recognized as an indicator of adaptation to the new reality of life with demands and limitation of one's state of health and effective psychological coping in a situation of the sudden collapse of one's capabilities and sense of security. On the other hand, a manifestation of PTSD symptoms and reduced quality of life may contribute to an increased risk of another heart attack and mortality (7, 8).

As defined by the World Health Organization (9), quality of life comprises an individual's assessment of physical health, emotional state, self-dependence, and interactions with the social environment. Meanwhile, chronic illness changes the functioning in all of these domains. The ability to adapt to new situations may be determined by a variety of factors such as temperamental characteristics and coping strategies (10).

The Regulatory Theory of Temperament by Strelau (11) is primarily focused on the formal characteristics of behavior involving energetic and temporal categories. Temperamental traits included in the energetic aspect are sensory sensitivity, emotional reactivity, endurance, and activity, whereas the temporal aspect covers briskness and perseverance. Sensory sensitivity refers to the ability to perceive even very subtle sensory stimuli. Emotional reactivity describes one's disposition to an intense response to emotionally charged stimuli. Endurance can be defined as the mental capacity to withstand prolonged or exhausting conditions, whereas activity is the propensity to engage in behaviors under circumstances of high stimulation. Briskness is the ability to react swiftly and fluently move from one behavior to another. Lastly, perseverance refers to the tendency to recurrently experience particular emotional states in response to stimuli, even when those stimuli are not present anymore. The functional importance of temperamental traits proposed in the Regulatory Theory of Temperament has been the focus of multiple pieces of research, a number of which was conducted on survivors of cataclysms and calamities. Traits such as emotional reactivity, perseverance, and activity have been found to be important moderators of the psychological implications (e.g., PTSD) of the trauma they have experienced (12). Furthermore, some studies imply that lower emotional reactivity might itself have a protective effect against symptoms of cancer-related trauma in adults (13, 14).

Thus, temperamental traits have the potential to function as moderators of the impact of life events by enhancing or reducing their stimulatory effect (15). Moreover, stress-related coping strategies might be influenced by them as well.

As already mentioned, after MI, patients are facing a number of different stress sources, primarily the disease itself but also mood swings, changes in their organisms and its capabilities, and those related to their personal, family, and professional life. So far, research indicates that quality of life is more influenced by the way one copes with stress than the stress itself (16). Indeed, individuals tend to vary in the range of coping strategies. Some are more likely to use a more task-oriented approach, whereas others would rather apply more emotion-oriented or avoidant strategies (17). It is worth noting that coping strategies, which may be regarded as ineffective in normal functioning, may yet prove beneficial under certain conditions. In fact, it has been reported that post-heart attack patients may actually feel better while adopting a more avoidant approach (18). However, further research is still needed in this area.

## Current Study

**Figure 1:**
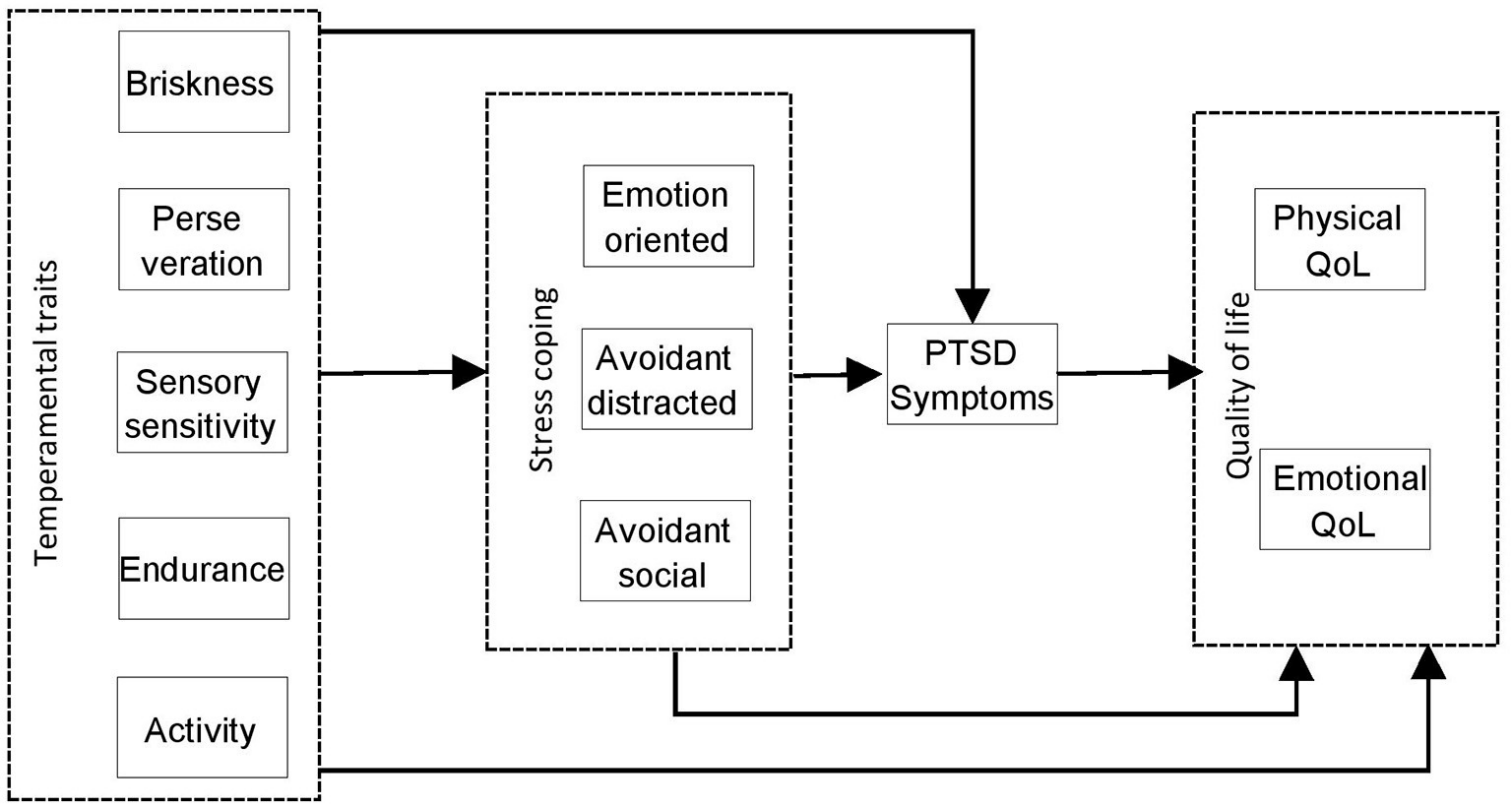
Preliminary model considering relationships between temperamental traits, stress coping styles, PTSD symptoms, and quality of life.

This paper aims to explore four different elements of coping with the circumstances after a heart attack, such as the trauma associated with a sudden breakdown in health and the changes required to promote recovery. Those elements comprise temperamental traits, stress coping styles, symptoms of PTSD, and quality of life. These are considered to be four consecutive phases in the psychological processes model. Based on this model, temperamental characteristics influence the way of coping with stress, which may appear more or less effective depending on the patient's situation. Subsequently, more or less adaptive strategies of coping with stress may either lead to the development of PTSD symptoms or adequate coping with the experienced trauma. As a result, quality of life is affected by one's ability to cope with a new situation. The subsequent stages of this process are temperamental characteristics, coping with stress, PTSD symptoms, and quality of life (see Figure 1).

Temperamental traits, which may directly or indirectly influence coping and all consecutive stages of the process, can only undergo relatively slow changes throughout time. Thus, coping flexibility after MI is restricted to the degree to which temperamental traits determine coping with stress.

The objective of the presented study is to verify the following research hypotheses:

H1. Temperamental traits are in relation to coping styles, presence of PTSD symptoms, and quality of life after MI.

H2. Stress coping styles are in relation to the presence of PTSD symptoms and quality of life.

H3. PTSD symptoms are in relation to the quality of life.

## Methods

The study included 96 participants who had suffered a MI. Of these, there were 51 women and 45 men aged 24–85 years (M = 52.27; SD = 12.29). All participants were survivors of ST-elevation MI with no record of other chronic diseases. They were tested in the hospital from 6 to 12 months after the incident. Participation in the study was voluntary and anonymous; no compensation was paid to participants.

Seventy of the participants (72.9%) were known to have children. In Table 1, the frequency distribution is shown for a place of residence, education level, marital status, and illness duration. The most common place of residence was a large city with a population of more than 100,000. Secondary school was the most common level of education. The majority of the participants were married.

**Table 1:** Frequency distribution—place of residence, educational level and marital status, and duration of illness.

**Place of residence**	***n***	**%**
City with number of inhabitants more than 100,000	47	49.0
City with number of inhabitants from 1,000 to 100,000	37	38.5
Small town with number of inhabitants < 1,000	12	12.5
Level of education	*n*	%
Higher completed	22	22.9
Higher professional	6	6.3
Post-secondary	14	14.6
Secondary	39	40.6
Occupational	14	14.6
Elementary	1	1.0
Marital status	*n*	%
Married	71	74.0
Informal relationship	13	13.5
Single	12	12.5
Duration of illness	*n*	%
< 1 year	48	50.0
1–2 years	30	31.3
Longer than 2 years	18	18.8

*n, number of participants; %, percentage of sample*.

The local Research Ethics Committee of the Faculty of Psychology of the University of Warsaw approved the project of this study.

To assess temperamental traits, the Formal Characteristics of Behavior—Temperament Inventory (19) was used. There are six temperament traits measured by this questionnaire: briskness, perseverance, sensory sensitivity, emotional reactivity, endurance, and activity. Overall, 120 items were included, 20 of them on each of the six scales. The range of pints that can be obtained on each scale is from 0 to 20. Participants are asked to answer affirmatively or contradictorily to each of the questionnaire items. The psychometric characteristics of the Formal Characteristics of Behavior—Temperament Inventory are good. Depending on the scale, Cronbach's α reliability ranges from 0.72 to 0.86. In the presented research, reliability coefficient values ranged from 0.60 to 0.70.

To assess stress coping style, the Coping Inventory for Stressful Situations, developed by Endler and Parker (17), was used. It covers a total of 48 diagnostic positions. Three stress coping styles measured by this questionnaire are distinguished, i.e., emotion-oriented, task-oriented, and avoidant. The latter is two-dimensional, with distraction as the first dimension and social diversion as the second one. Cronbach's α reliability for the Polish adaptation ranges from 0.71 to 0.92. In the current study, reliability coefficient values ranged from 0.70 to 0.74 (20).

Furthermore, the PTSD Inventory created by Strelau et al. (21) was used to measure the intensity of PTSD symptoms. Using this questionnaire, PTSD symptoms can be quantitatively assessed based on the following dimension: intrusiveness/hyperactivity (I/H) (recurring thoughts related to the experienced trauma that cause increased arousal) and avoidance/numbness (A/N) (avoidance and impaired response to the stimuli associated with the traumatic event). A general scale of PTSD symptoms is included as well. The inventory consists of 30 items rated on a four-point scale from 1 (symptom never present) to 4 (symptom constantly present). Both the general scale and two subscales show good reliability, with Cronbach's α ranging from 0.90 to 0.97. In the present study, with the instruction referring to the endured disease as a traumatic experience, the reliability coefficient values were 0.70 for intrusiveness/hyperarousal and 0.75 for avoidance/numbness.

As for measuring the quality of life, the 36-item Short Form Survey questionnaire in its Polish version (22) was applied. It consists of 36 positions referring to the state of health and reaction to illness. Two main dimensions of the quality of life are taken into account. The first one, namely physical quality of life, covers somatic aspects such as physical activities and pain and its impact on daily functioning, whereas the second one is an emotional quality, which includes issues such as social activity, the emotional consequences of health-related limitations, energy level, and fatigue. Furthermore, apart from these dimensions, the questionnaire also provides an overall measure of the quality of life. A five-point scale is used for the answers. In the Polish version of the questionnaire, the score is inversely proportional to the quality of life, which means that a higher score indicates a low quality of life, whereas a lower score corresponds to a high quality of life. The questionnaire is characterized by good reliability, with Cronbach's α ranging from 0.75 to 0.95. In the present study, the reliability coefficient values were 0.72 for physical quality of life and 0.86 for emotional quality of life.

## Data Analysis

Statistical analysis was conducted using IBM SPSS 24, and IBM AMOS 24 statistical package was used (23).

At first, descriptive statistics were calculated for the demographic characteristics of the sample and the interval scales. Secondly, an analysis of the relationship between quality of life and the sample characteristics was conducted.

The main analysis consisted of two phases. In a first step, the dependencies between the consecutive concepts of the assumed model were examined using regression analysis, starting with the quality of life and moving backward to temperamental traits. With the obtained regression coefficients, it became possible to build a model, subsequently tested by conducting a path analysis. In line with the results of the prior regression analysis, the input model comprised pathways that were found to be statistically significant.

## Results

Descriptive statistics of all interval scales, i.e., mean values, standard deviation, minimum and maximum values, are presented in Table 2.

**Table 2:** Descriptive statistics for interval scales.

**Questionnaires**	**Variables**	**M**	**SD**	**min**	**max**
FCB-TI	Briskness	11.24	3.48	1	19
	Perseverance	10.26	3.45	1	20
	Sensory sensitivity	10.43	3.52	3	20
	Emotional reactivity	10.55	3.23	3	18
	Endurance	8.18	3.46	0	16
	Activity	9.96	3.85	1	18
CISS	Task-oriented coping	50.48	7.17	37	72
	Emotion-oriented coping	46.47	7.32	23	69
	Avoidant coping	48.15	6.30	27	63
	Avoidant-distracted coping	23.43	4.21	10	30
	Avoidant-social coping	15.76	2.60	9	23
PTSD-C	Intrusion/arousal	34.45	5.30	15	44
	Avoidance/numbing	33.59	6.20	18	45
	PTSD-C Total score	68.04	10.62	33	82
SF-36	Physical Qol	54.86	11.14	24	84
	Emotional Qol	28.67	6.49	11	56
	Quality of life index	83.53	15.86	35	136

*M, mean; SD, standard deviation; min, minimum; max, maximum*.

No significant difference in the quality of life was observed between men and women. The Mann–Whitney *U*-test results were statistically insignificant for both physical quality of life, U = 1,135.50, *p* > 0.05, and emotional quality of life, U = 890.50, *p* > 0.05.

The Spearman rank correlation coefficient was applied to explore the correlations between quality of life, disease duration, and demographic variables, i.e., level of education and size of the place of residence.

Results indicate that physical quality of life correlates positively with illness duration, ρ(*n* = 96) = 0.206, *p* < 0.05. However, no significant correlation was observed either with place of residence ρ(*n* = 96) = 0.009, *p* > 0.05, or level of education, ρ(*n* = 96) = −0.194, *p* > 0.05.

The emotional quality of life, on the other hand, correlated negatively with size of place of residence ρ(*n* = 96) = −0.247, *p* < 0.05, and level of education, ρ(*n* = 96) = −0.252, *p* < 0.05, whereas no significant correlation was observed with illness duration, ρ(*n* = 96) = 0.201, *p* > 0.05.

Relationships between consecutive model stages were first analyzed using regression analysis. PTSD symptoms were analyzed as an explanatory variable for both physical and emotional quality of life, coping styles were analyzed as an explanatory variable for intrusion and avoidance PTSD symptoms, and temperamental traits were analyzed as an explanatory variable for coping styles. The variables used are of univariate distribution; thus, the assumption of normality of distribution is not met. Under these circumstances, the analysis was based on bootstrap samples. For the purpose of the study, 1,000 such samples were generated. Estimation of the model was carried out assuming a 95% confidence interval for the parameters. The obtained coefficients are presented in Table 3.

**Table 3:** Estimates from regression models for subsequent stages.

**Physical Qol**	**Beta**	**SE**	**95% CI**	***P*-value**
Intrusion/arousal	−0.13	0.18	−0.48, 0.23	0.485
Avoidance/numbing	0.43	0.15	0.15, 0.71	0.001
Emotional Qol	Beta	SE	95% CI	*P*-value
Intrusion/arousal	0.13	0.15	−0.15, 0.44	0.388
Avoidance/numbing	0.24	0.18	−0.12, 0.58	0.201
Avoidance/numbing	Beta	SE	95% CI	*P*-value
Task-oriented coping	−0.14	0.11	−0.34, 0.08	0.186
Emotion-oriented coping	0.33	0.11	0.10, 0.54	0.004
Avoidant-distracted coping	0.22	0.10	−0.01, 0.41	0.036
Avoidant-social coping	−0.21	0.10	−0.41, 0.01	0.046
Emotional Qol	Beta	SE	95% CI	*P*-value
Task-oriented coping	−0.12	0.14	−0.38, 0.15	0.399
Emotion-oriented coping	0.14	0.14	−0.16, 0.38	0.319
Avoidant-distracted coping	−0.23	0.11	−0.47-, 0.03	0.042
Avoidant-social coping	−0.30	0.13	−0.56-, 0.07	0.029
Emotion-oriented coping	Beta	SE	95% CI	*P*-value
Briskness	−0.22	0.15	−0.52, 0.05	0.168
Perseverance	0.34	0.13	0.07, 0.59	0.016
Sensory sensitivity	−0.29	0.12	−0.50-, 0.05	0.020
Emotional reactivity	0.03	0.10	−0.20, 0.21	0.817
Endurance	0.32	0.11	0.10, 0.52	0.005
Activity	0.06	0.14	−0.21, 0.36	0.656
Avoidant-distracted coping	Beta	SE	95% CI	*P*-value
Briskness	−0.14	0.14	−0.43, 0.09	0.329
Perseverance	0.27	0.11	0.04, 0.49	0.024
Sensory sensitivity	−0.11	0.12	−0.32, 0.13	0.336
Emotional reactivity	−0.02	0.13	−0.27, 0.23	0.917
Endurance	0.25	0.11	0.04, 0.49	0.036
Activity	0.33	0.12	0.08, 0.55	0.009
Avoidant-social coping	Beta	SE	95% CI	*P*-value
Briskness	0.31	0.13	0.05, 0.58	0.026
Perseverance	0.22	0.11	0.02, 0.47	0.055
Sensory sensitivity	−0.02	0.12	−0.27, 0.22	0.875
Emotional reactivity	−0.05	0.11	−0.27, 0.16	0.651
Endurance	0.08	0.11	−0.11, 0.30	0.411
Activity	0.21	0.11	−0.02, 0.43	0.067

*Beta, standardized regression coefficient; SE, standard error; 95% CI, bootstrap confidence interval*.

A significant association was observed between avoidance/numbing and physical quality of life. Higher scores on the 36-item Short Form Survey questionnaire were associated with higher levels of disability, whereas higher levels of avoidance/numbness were accompanied by stronger deterioration in physical quality of life.

However, the emotional quality of life was found not to be related to the level of PTSD symptoms, nor avoidance/numbness or intrusions/arousal. Therefore, in the subsequent regression analysis, coping styles were analyzed as explanatory variables.

It was found that higher levels of both avoidant-distracted and avoidant-social and coping were related to a lower level of the emotional quality of life. Furthermore, it was also shown that both emotion-oriented and avoidant-distracted coping was positively related to avoidance/numbness. Contrastingly, a negative association was observed between avoidance/numbing and avoidant-social coping.

Moreover, higher perseverance and endurance and lower sensory sensitivity were found to be related to the more frequent application of emotion-oriented coping. On the other hand, higher perseverance and endurance along with higher activity were shown to be predicates of a tendency to use avoidant-distracted coping more frequently. Finally, more frequent use of avoidant-social coping was found to be predicted by a higher level of briskness.

**Figure 2:**
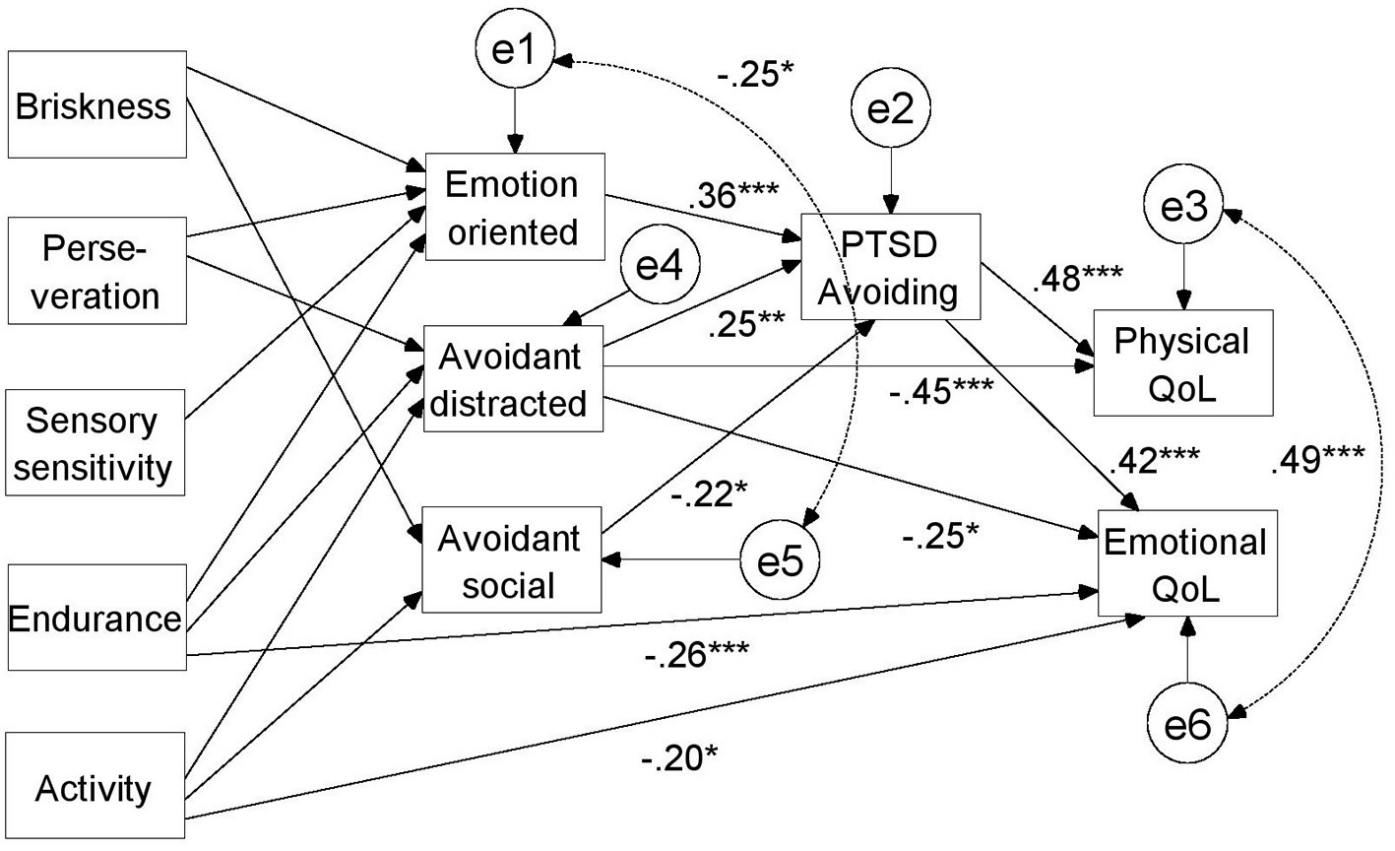
Final model of associations between temperamental traits, stress coping styles, PTSD symptoms, and quality of life with standardized regression coefficients.

After that, in the second stage of statistical analysis, only statistically significant explanatory variables were included. A path analysis, also based on 1,000 bootstrap trials, was used to examine the final model. The results of this analysis are shown in Figure 2.

Based on modifying indicators with a threshold value of 4, paths were added between the physical quality of life and avoidant-distracted coping, between the emotional quality of life and avoidance/numbing, between the emotional quality of life and endurance, between the emotional quality of life and activity, between emotion-oriented coping and briskness, and between avoidant-social coping and activity. As the path between the emotional quality of life and avoidant-social coping after inclusion of endurance and activity appeared to be statistically insignificant, it was excluded from further analysis.

For more transparency, the regression coefficients between temperamental traits and stress coping styles are given in Table 4.

**Table 4:** Regression weights between temperamental traits and stress coping styles acquired in path analysis.

**Stress coping styles**	**Temperamental traits**	**Beta**
Emotion-oriented coping	Perseverance	0.38^***^
Emotion-oriented coping	Sensory sensitivity	−0.29^***^
Emotion-oriented coping	Endurance	0.31^***^
Avoidant-distracted coping	Perseverance	0.22^*^
Avoidant-distracted coping	Endurance	0.20^*^
Avoidant-distracted coping	Activity	0.31^***^
Avoidant-social coping	Briskness	0.34^***^
Avoidant-social coping	Activity	0.22^*^
Emotion-oriented coping	Briskness	−0.21^*^

* *p < 0.05;*

*** *p < 0.001*.

The final fit of the model proved to be satisfactory, χ^2^(32, *N* = 96) = 39.34, *p* > 0.05, *CFI* = 0.97, *TLI* = 0.94, *RMSEA* = 0.05, [90% *CI* = 0.01, 0.10].

It was shown that a lower level of briskness, higher level of perseverance, lower level of sensory sensitivity, and a higher level of endurance were associated with a tendency toward emotion-oriented coping. The latter, in turn, was found to correlate positively with avoidance/numbing.

A higher level of perseverance, endurance, and activity was, on the other hand, related to a higher level of avoidant-distracted coping, which was also shown to correlate positively with avoidance/numbing.

Furthermore, a higher level of briskness and activity appeared to be related to a more avoidant-social coping style, being in turn negatively correlated with avoidance/numbness level. Moreover, avoidance/numbing level was also negatively correlated with both the emotional and physical qualities of life. On the other hand, a higher level of avoidant-distracted coping was directly related to a better physical quality of life; meanwhile, a higher level of endurance and activity was associated with a better emotional quality of life.

## Discussion

Through the conducted analysis, a complex model was obtained showing multiple associations between the different psychological variables, hence the main conclusion that quality of life depends on a number of factors. Considering the temperamental profile, it can be stated that only two traits unambiguously explain a better quality of life. Namely, higher sensory sensitivity is related to a lower level of emotion-oriented coping, which in turn may lead to a reduction in PTSD symptoms and thus improved physical and emotional quality of life. Furthermore, a higher level of briskness is associated with both a lower level of emotion-oriented coping and a higher level of avoidant-social coping, which is, in turn, explanatory for milder PTSD symptoms and consequently higher quality of life.

As previously outlined, high sensory sensitivity enables one to detect and perceive even low-level stimuli (11). As such, it has the potential to induce openness to experience and appreciation of small details in the environment, making emotional coping unnecessary and thus enhancing life satisfaction. Meanwhile, briskness is the foundation for increased adaptive capacity, potentially allowing for better adaptation to the disease-related limitations.

However, the relevance of other temperamental traits appears to be more ambiguous. In stress coping, both emotion-oriented and avoidant-social coping styles are clearly related to the higher level of PTSD symptoms, which in turn explain the lower physical and emotional quality of life. However, it is important to note that avoidant-distracted coping is associated not only with better quality of life—both physical and emotional, but also with a higher level of PTSD symptoms, which, by contrast, are linked to lower quality of life. A possible explanation is that avoidance-distraction coping may be associated with a higher quality of life, provided that it is a strategy of coping with the disease and its consequences and not a strategy of coping with the trauma itself. Nevertheless, further research is still much needed on the subject, taking into account also other variables. According to a study by Ayers et al. (24), dysfunctional coping strategies were associated with PTSD after MI, whereas the findings presented by Marke and Bennett (25) indicated that problem- and emotion-focused coping styles were related to a lower likelihood of developing PTSD.

Moreover, higher perseverance and endurance are associated with a tendency to more emotion-oriented coping, thereby explaining the lower quality of life, and yet, at the same time, higher perseverance is related to more intense avoidant-distracted coping, which may or may not be beneficial to the quality of life. Furthermore, higher activity is connected with more avoidant-social coping, linked to a better quality of life; however, it may also be associated with more avoidant-distracted coping. Although a higher level of activity, coming with a higher need for stimulation can be of some benefit; simultaneously, it may prove maladaptive while dealing with cardiovascular disease.

Besides, endurance and activity are also directly related to the emotional quality of life, as it appears that higher intensities of these characteristics are likely to result in a more positive impact. Due to their immunizing aspects, both of these characteristics may directly contribute to one's life satisfaction level.

According to the findings of several studies (26, 27), higher neuroticism has been shown to contribute to higher severity of PTSD symptoms after MI. Although temperamental traits taken into account in the described study are more specific with stricter definitions than neuroticism as a personality dimension, in theory, neuroticism is believed to be associated with high emotional reactivity, low endurance, and high perseverance. Such connections have also been demonstrated in empirical studies (28). Nevertheless, the findings of the current study failed to confirm a disadvantageous role of emotional reactivity. It is also important to note that, depending on which stress coping component is considered, perseverance and endurance may or may not prove to be of benefit.

With the findings of the current study, however, the significant influence of PTSD symptoms on quality of life is confirmed. In a number of previous studies, both retrospective and prospective, negative effects of PTSD symptoms have been reported (29–31). Similarly, the model presented in this paper highlights this negative relationship, although PTSD symptom level was analyzed as an implication of coping styles, i.e., emotion-oriented coping may be associated with an increased level of anxiety and tension, both being symptomatic of posttraumatic disorders. Consequently, suffering posttraumatic symptoms along with the underlying effects and limitations resulting from cardiovascular disease can substantially undermine one's ability to enjoy life.

Undoubtedly, the findings of the current study support a model of psychological processes where the consecutive stages are temperament, coping styles, PTSD, and quality of life. Nevertheless, there were certain limitations to this study. Presumably, the most significant was the lack of longitudinal data. Data obtained during the adaptation process with the controlled amount of time after infarction might have strengthened the conclusion. Moreover, it is important to note that there was no control group in this study. Thus, the obtained results apply only to the clinical group of patients after MI. Finally, one may also argue that there is a need to monitor health status variables in many aspects other than cardiovascular disease, including the healthcare standard along with its implications, and similarly the social factors, e.g., the support provided to post-MI patients by their families and loved ones (10, 32).

## Conclusions

In the presented research, relationships between four different aspects of coping with the situation of MI were analyzed. These aspects were temperamental traits, stress coping styles, presence of PTSD symptoms, and quality of life. Although the obtained model suggests that the quality of life after MI depends on many factors, the conducted analysis has shown several more significant correlations. Namely, emotion-oriented coping is correlated with sensory sensitivity, briskness (negatively), perseverance, endurance, and level of PTSD symptoms (positively). Avoidant-social coping is correlated with briskness, activity, and level of PTSD symptoms, whereas avoidant-distracted coping is correlated with perseverance and activity. Furthermore, it has been confirmed that PTSD symptoms may influence the quality of life. There were, however, certain limitations to this study, such as the lack of a control group or longitudinal data. It would be beneficial to include it in future research.

## Data Availability Statement

The raw data supporting the conclusions of this article will be made available by the authors, without undue reservation.

## Ethics Statement

The studies involving human participants were reviewed and approved by Ethical committee of the Faculty of Psychology, University of Warsaw. The patients/participants provided their written informed consent to participate in this study.

## Author Contributions

The idea of the research was developed jointly. The research was conducted by AB. MW supported the process of obtaining data as far as possible and made sure that all answers in the questionnaires were marked. In the event of missing responses, MW made an attempt to complete the missing responses and entered the results into the database and dealt with the preliminary statistics. AB wrote an article, made corrections, and made proofreading. Both authors contributed to the article and approved the submitted version.

## Funding

This work was supported by the Faculty of Psychology of the University of Warsaw and by the IDUB (grant no: IDUB PSP 501-D125-20-0001241).

## Conflict of Interest

The authors declare that the research was conducted in the absence of any commercial or financial relationships that could be construed as a potential conflict of interest.

## Publisher's Note

All claims expressed in this article are solely those of the authors and do not necessarily represent those of their affiliated organizations, or those of the publisher, the editors and the reviewers. Any product that may be evaluated in this article, or claim that may be made by its manufacturer, is not guaranteed or endorsed by the publisher.
